# Bemotrizinol and the physiology of photoprotection: a new opportunity to reduce cutaneous ultraviolet damage

**DOI:** 10.1152/function.90019.2026

**Published:** 2026-12-01

**Authors:** Phillip Holler, George Cotsarelis

**Affiliations:** ^1^Department of Dermatology, University of Pennsylvania Health System, Philadelphia, Pennsylvania, United States; ^2^Department of Dermatology, Perelman School of Medicine, University of Pennsylvania, Philadelphia, Pennsylvania, United States

Ultraviolet (UV) radiation is a near-universal environmental driver of injury to the genomic integrity, immune surveillance, and barrier function of the skin and acts as the primary cause of cutaneous malignancy, including melanoma, in the United States (1–4). As such, the recent US Food and Drug Administration (FDA) approval of bemotrizinol, a photostable broad-spectrum UV chemical filter, represents an exciting new development for United States consumers and health care providers seeking to curb the cumulative burden of UV radiation on human health (5). Used for over 30 years in more than 50 countries in Europe and Asia, bemotrizinol (marketed under the brand name PARSOL Shield) combines durable UV spectral coverage with minimal systemic penetration (5, 6). In addition to these favorable photoprotective and safety characteristics, sunscreens containing bemotrizinol have earned a reputation as pleasant to apply and wear, translating to more consistent patient use (6, 7). Thus, bemotrizinol provides a prime example of chemical innovation encompassing cutaneous biology, topical delivery, and real-world behavior.

Skin damage from sunlight reflects the distinct but overlapping effects of ultraviolet B and ultraviolet A radiation (2, 3). Short-wavelength UVB photons, the main cause of sunburn, are strongly associated with direct DNA photoproduct formation (thymine dimers, cytosine dimers, etc.) and mutational events in epidermal keratinocytes (2, 3). Longer-wavelength, lower-energy UVA photons, on the other hand, penetrate more deeply into the dermis, where they produce reactive oxygen species, thereby fostering mitochondrial stress, matrix metalloproteinase activation, and collagen fragmentation (2, 3). UVA irradiation of the skin also leads to pigmentary dysregulation and immune dysfunction (2, 3, 7). Together, these processes link environmental UV exposure to photocarcinogenesis, inflammation, photoaging, dyspigmentation, and impaired tissue repair (2–4, 7). Effective photoprotection, therefore, does not only involve the prevention of acute sunburn. Rather, the goal is to reduce repeated molecular and cellular insults that chronically accumulate throughout the epidermis and dermis (1, 4, 7).

The physiological relevance of a sunscreen chemical filter depends not only on the wavelengths it absorbs at application, but also on whether that protection persists during exposure (4, 6). Historically, United States chemical sunscreens have relied heavily on avobenzone for broad UVA coverage, despite its tendency to rapidly degrade unless stabilized by other ingredients (5, 6). In contrast, bemotrizinol absorbs strongly across both UVA and UVB wavelengths and is highly photostable (5, 6). In practical terms, photostability helps to preserve and prolong the protective activity, and at the same time, serves to stabilize accompanying UV chemical filters within the same sunscreen formulation (5, 6). This is important because intermittent loss of UVA protection may leave dermal structures exposed to oxidative injury even when a sunscreen nominally qualifies as broad spectrum (2–4).

The approval of any new UV filter must consider the physiology of the stratum corneum, the outermost layer of the skin, which functions as both a defensive barrier and the main determinant of topical bioavailability (2, 3). Public concern about chemical sunscreens has intensified recently as studies have shown that older components such as oxybenzone and octinoxate can be detected in blood after repeated application, although the clinical significance of those findings remains uncertain (8, 9). Bemotrizinol differs from many earlier chemical filters due to its large molecular size and low tendency for meaningful percutaneous penetration (5, 6). FDA-reviewed testing found minimal systemic absorption, with measured concentrations remaining well below the agency’s threshold for additional systemic safety evaluation (5, 6). This profile suggests that its principal physiological action is to remain local, at the skin surface and upper barrier interface, where UV photons are absorbed before initiating downstream tissue injury and carcinogenesis (2–6). In addition, rigorous clinical testing demonstrated that bemotrizinol is nonmutagenic, does not disrupt the endocrine system, and rarely irritates the skin (5, 6).

A sunscreen’s biological promise is realized only when it is applied adequately and repeatedly (1, 4, 7). This is where cosmetic formulation becomes a physiological issue rather than a marketing detail. Products that leave an opaque residue, feel heavy, or interfere with makeup or facial hair are less likely to be used consistently, especially across diverse skin tones and daily routines (7). Because bemotrizinol dissolves into cosmetic oils and can be incorporated into lighter, transparent formulations, it may help narrow the gap between laboratory efficacy and real-world protection (6, 7). The relevant outcome is not merely cosmetic elegance for its own sake, but improved patient compliance. Greater adherence reduces cumulative UV exposure and thereby improves the long-term trajectory of photoaging, inflammation, pigmentary change, and skin cancer risk (1, 4, 7).

The approval of bemotrizinol also highlights the role of regulatory science in determining which physiological interventions reach patients and populations (5). For decades, United States sunscreen innovation was constrained by a limited set of approved active ingredients, even as international markets adopted newer photostable filters (5). Recent reforms to the over-the-counter monograph process and renewed attention from dermatology and public-health advocates created a clearer pathway for evaluating international safety data and modern absorption studies (5, 9). This history is instructive. The translation of a protective mechanism into population benefit requires not only molecular efficacy but also regulatory frameworks capable of distinguishing theoretical uncertainty from clinically meaningful risk (4, 5).

The availability of bemotrizinol should not be interpreted as a single technological solution to the public health burden of ultraviolet injury in the United States (1, 4, 7). Ideal photoprotection will continue to require behavioral measures, protective clothing, sun avoidance, equitable access, and sustained education about regular and proper sunscreen use (1, 4, 7). Nevertheless, the approval of this highly effective and safe UV filter represents an opportunity to reassert sunscreen as a translational intervention (4–6). Its importance lies at the intersection of mechanism and behavior. Better spectral durability will reduce the skin’s exposure to UV damage, while more acceptable formulations will encourage patient compliance with recommended sunscreen use (1, 5, 7). Future work should assess not only absorption and toxicology but also whether products containing newer filters such as bemotrizinol improve adherence, reduce biomarkers of oxidative and DNA damage, and afford clinically meaningful outcomes to decrease skin disease across diverse populations (2–7).
